# Hypoxia-Driven TGFβ Modulation of Side Population Cells in Breast Cancer: The Potential Role of ERα

**DOI:** 10.3390/cancers15041108

**Published:** 2023-02-09

**Authors:** Paraskevi Mallini, Miaojuan Chen, Kamilla Mahkamova, Thomas W. J. Lennard, Yue Pan, Dan Wei, Katherine Stemke-Hale, John A. Kirby, Gendie E. Lash, Annette Meeson

**Affiliations:** 1 Biosciences Institute, International Centre for Life, Newcastle University, Central Parkway, Newcastle upon Tyne NE1 3BZ, UK; 2 Guangzhou Institute of Pediatrics, Guangzhou Women and Children’s Medical Center, Guangdong Provincial Clinical Research Center for Child Health, Guangzhou Medical University, Jinsui Road, Tianhe, Guangzhou 510623, China; 3 Northern Institute for Cancer Research, Newcastle University, 3rd Floor William Leech Building, Framlington Place, Newcastle upon Tyne NE2 4HH, UK; 4 Department of Systems Biology, MD Anderson Cancer Center, University of Texas, Houston, TX 77030, USA; 5 Translational and Clinical Research Institute, Faculty of Medical Sciences, Newcastle University, 3rd Floor William Leech Building, Framlington Place, Newcastle upon Tyne NE2 4HH, UK

**Keywords:** breast cancer stem cells, cytokines, CoCl_2_, TGFβ, side population cells, 4-hydroxytamoxifen, hypoxia

## Abstract

**Simple Summary:**

A process termed epithelial-to-mesenchymal transition can allow cancer cells to acquire or increase stem cell-like characteristics, which include increased potentials for migration and tissue invasion. This study focused on the effects of transforming growth factor-beta or hypoxia, which are both drivers of epithelial-to-mesenchymal transition, on rare, stem cell-like “side population” cells within the well-characterised human breast cancer cell lines MDA-MB-231 (a model for hormone non-responsive breast cancer) and MCF7 (a model for hormone responsive breast cancer). Both cell lines responded similarly to transforming growth factor-β, whereas hypoxia decreased the side-population cells in MDA-MB-231 but increased the cells in MCF7. Further work performed at the single-cell level, showed that hypoxia specifically increased multi-drug resistance of MCF7 side population cells. Taken together, these data suggest that a better understanding of the regulation of epithelial-to-mesenchymal transition is needed and might allow the identification of new therapeutic targets for the treatment of breast cancer.

**Abstract:**

Epithelial-to-mesenchymal transition (EMT) is known to be important in regulating the behaviour of cancer cells enabling them to acquire stem cell characteristics or by enhancing the stem cell characteristics of cancer stem cells, resulting in these cells becoming more migratory and invasive. EMT can be driven by a number of mechanisms, including the TGF-β1 signalling pathway and/or by hypoxia. However, these drivers of EMT differ in their actions in regulating side population (SP) cell behaviour, even within SPs isolated from the same tissue. In this study we examined CoCl_2_ exposure and TGF-β driven EMT on SP cells of the MDA-MB-231 and MCF7 breast cancer cell lines. Both TGF-β1 and CoCl_2_ treatment led to the depletion of MDA-MB-231 SP. Whilst TGF-β1 treatment significantly reduced the MCF7 SP cells, CoCl_2_ exposure led to a significant increase. Single cell analysis revealed that CoCl_2_ exposure of MCF7 SP leads to increased expression of ABCG2 and HES1, both associated with multi-drug resistance. We also examined the mammosphere forming efficiency in response to CoCl_2_ exposure in these cell lines, and saw the same effect as seen with the SP cells. We suggest that these contrasting effects are due to ERα expression and the inversely correlated expression of TGFB-RII, which is almost absent in the MCF7 cells. Understanding the EMT-mediated mechanisms of the regulation of SP cells could enable the identification of new therapeutic targets in breast cancer.

## 1. Introduction

Progression of breast cancer to a metastatic disease relies in part on tumour cells at the primary tumour site becoming more migratory and invasive. This process is mediated by epithelial-to-mesenchymal transition (EMT) and is followed by the reverse phenomenon, mesenchymal-to-epithelial transition (MET) which enables new tumours to be established.

It is now accepted that cancer stem cells (CSCs) are important in disease progression. Their abilities for self-renewal, apoptosis resistance and efflux help to enable tumour regrowth. This ability of CSCs to efflux chemotherapeutic drugs, amongst other compounds, is primarily due to the expression of members of the ABC transporter family [1]. Solute transporters may also have a role in the efficacy of chemotherapeutic treatments as substrates for several of these transporters are anticancer drugs [2]. Side population (SP) cells can be isolated based on their ability to expel vital dyes, through expression of ABC transporters [3]. SP cells have been found in both mouse and human breast tissue and have been shown to generate epithelial and luminal cells and structures in vitro and in vivo, [4]. Increased SP cell prevalence has been associated with triple negative or hormonal non-responsive breast cancer patients and the presence of SP cells may be responsible for their poor clinical outcome [5].

EMT is thought to contribute to the regulation of breast CSCs. Human mammary epithelial cells (HMLE) induced to undergo EMT gave rise to an increased percentage of CD44+/CD24- cells which had stem cell characteristics [6]. In contrast, FOXC2 knockdown in EMT-induced HMLE cells led to the generation of cells with epithelial-like properties, and a decrease in CD44+/CD24- cells [7]. 

While it is known that EMT is regulated by several growth factors and cytokines, one important signalling pathway is mediated by TGF-β. In breast cancer TGFB-RII expression is inversely correlated with ER expression [8] and the CD44+/CD24- cell phenotype has been associated with ER-/TGFB-RII+ patients [9]. This suggests that ER status and the presence of a functional TGF-β signalling pathway can determine the regulation of putative breast CSCs through EMT in different breast cancer patients. 

TGF-β treatment appears to have a regulatory effect on SP cells. In human diffuse-type gastric carcinoma cells downregulation of ABCG2 resulted in reduced SP cell numbers [10]. While TGF-β1 treatment of the MCF7 breast cancer cell line eliminated the SP population, decreased ABCG2 expression and reduced cell viability in the presence of mitoxantrone. These effects were reversable on removal of TGF-β1 [11]. In addition, TGF-β1 treatment of thyroid cancer cell lines resulted in a reduction in ABCG2 mRNA and a reduction in SP cell numbers, while removal of endogenous TGF-β1 resulted in restoration of the SP cells [12]. Exposure of ovarian cancer cell lines to TGF-β1 resulted in reduced SP cell numbers and an increase in some markers of EMT [13].

Hypoxia has also been reported to drive EMT in cancer cells [14,15]. Hypoxia-induced EMT has resulted in the acquisition of mesenchymal properties and the upregulation of E-cadherin repressors [16,17]. Hypoxia also seems to stimulate the activation of known EMT-related pathways, including TGF-β, Notch, Wnt and Hedgehog [16,18,19].

Hypoxia is also thought to have a positive regulatory impact on SP cells. Hypoxia and re-oxygenation led to an increase in ABCG2 expression in murine kidney SP cells and protection against hypoxic damage [20]. In thyroid cancer cell lines, treatment with CoCl_2_ led to a significant increase in SP cell numbers in two thyroid cancer cell lines [21]. While HIF-2α expression has also been associated with high ABCG2 expression, histology-grade and Ki67 expression in invasive human breast cancer, suggesting that HIF-2α could be a reliable prognostic marker for development of drug resistance and metastasis in breast cancer [22].

This study was designed to determine the effects of EMT through ΤGF-β1 and CoCl_2_ treatment on the SP population in the MDA-MB-231 (ER-/PR-/HER2-) and MCF7 (ER+/PR+/HER2-) cell lines. The impact of TGF-β exposure on the MCF7 SP cells alone was also confirmed in this study. Insight into the mechanisms that are involved in the regulation of these cells depending on breast cancer subtype could provide the basis of promising and more effective therapeutic strategies for the prevention of metastasis and drug resistance.

## 2. Materials and Methods

### 2.1. Cell Culture Conditions and Maintenance

MDA-MB-231 (validated by short tandem repeat (STR) DNA fingerprinting prior to being supplied by MD Anderson) and MCF7 (authenticated by STR finger printing by European Collection of Authenticated Cell Culture (ECACC) prior to purchase) were cultured in complete DMEM (cDMEM) (Sigma-Aldrich, St Louis, MI, USA) without phenol red, supplemented with 10% Foetal Bovine Serum (FBS) (Lonza, Basel, Switzerland), 2 mM L-glutamine (Sigma-Aldrich), 100 IU/mL penicillin, 100 μg/mL streptomycin (Life Technologies, Paisley, UK). The cells were maintained under standard oxygen conditions and medium was changed every 4–5 days. Cells were split 4:1 for protein and mRNA analysis.

### 2.2. TGF-β Induced EMT

1 × 10^5^ cells were seeded in 100 mm dishes. At 24 h cells were treated with TGF-β1 (R & D Systems, Abingdon, UK) 5 ng/mL for MDA-MB-231 and 10 ng/mL MCF7 for 3 days. As controls cells were treated in parallel without the addition of TGF-β1, 5 μΜ SB-505124 (Sigma-Aldrich) was added 30 min prior to TGF-β1 treatment to separate cells cultured under the same conditions and to confirm the inhibition of TGF-β1. After 72 h cells were harvested for further analysis.

### 2.3. CoCl_2_ Induced EMT

A total of 2 × 10^5^ cells were plated in 100 mm dishes. At 24 h cells were treated with 400 μM CoCl_2_ (Sigma-Aldrich). Controls were untreated cells. After 48 h cells were harvested for further analysis and culture medium was collected for ELISA assay.

### 2.4. Hypoxia Treatment

Cells were either plated in NuncTM Lab-TekTM II chamber slides (ICC) or six-well plates (qPCR). After 24 h the cells were placed in a Whitley H35 Hypoxystation set to an oxygen concentration of 3%. Controls were untreated cells. After 48 h, cells were harvested for further analysis and culture medium was collected for ELISA assay.

### 2.5. Treatment of MCF7 Cells with 4-Hydroxytamoxifen (Tam)

A total of 3 × 10^5^ cells were plated in 100 mm dishes and 1.5 or 2.5 μM Tam (Sigma-Aldrich) were added to the cell culture media at the time of plating. 24 h later cells were treated with CoCl_2_. Untreated cells and cells treated with 1.5 and 2.5 μM 4-hydroxytamoxifen were used as controls. After 48 h cells were harvested for SP analysis.

### 2.6. Flow Cytometry

Cells were trypsinized and diluted to give a final concentration of 1 × 10^6^ cells/mL in cDMEM. A total of 5 μL of DNase (Life Technologies) was added to each tube to prevent cell clumping. Additionally, 5 μL of fumitremorgin C (FTC,10 µM; Axxora, NY, USA) was added to one tube containing 1 × 10^6^ cells/mL. All cell samples were incubated at 37 °C using a MACsMix rotor (Miltenyi Biotec, Surrey, UK) for 15 min. All cell samples were then treated with Hoechst 33342 dye (Sigma-Aldrich), 5 μg/mL for MDA-MB-231 and 7 μg/mL for MCF7. The same concentrations of Hoechst 33342 were also added to the tubes containing FTC. For all SP experiments FTC was used as a control to confirm the SP phenotype. All cell samples were then incubated at 37 °C in the dark using a MACsMix rotor for 90 min, after which cells were washed in ice-cold 1X PBS and centrifuged at 3000 rpm for 5 min. Cells were re-suspended in 500 μL ice-cold 1X PBS and filtered through 70 μm cell strainers (BD Biosciences, Oxford, UK) into FACs tubes (BD falcon, Oxford, UK). All tubes were maintained on ice in the dark prior to cell analysis. Non-viable cells were excluded by the addition of 2 μL propidium iodide (PI; 2 μg/mL) (Sigma-Aldrich). A LSRII flow cytometer (BD Biosciences) was used for SP assays and data analysis was performed using FACS Diva software.

### 2.7. Mammosphere Forming Assay

Mammosphere formation efficiency was assessed using a modified protocol [23]. Briefly, cells were treated with CoCl_2_ as described above, detached when at 70–80% confluency, resuspended in PBS and counted. A 25 G needle was used to ensure that a single cell suspension was formed. A total of 1 × 10^5^ MCF7 and 1.5 × 10^5^ MDA-MB-231 cells were plated in low-adherent six-well plates containing 2 mL of mammosphere media. Cells were incubated in a humidified atmosphere at 37 °C and 5% CO_2_ for 9 days without moving the plates. Mammospheres with size greater than 50 μM were counted on the 9th day of culture using 5× magnification. Data generated are representative of n = 3 individual experiments for each cell line.

### 2.8. Immunocytochemistry

Cells were seeded in chamber slides or 6-well plates containing sterilized glass coverslips and cultured in cDMEM. When confluent, or after TGF-β1, CoCl_2_ or hypoxia treatment, cells were fixed in cold methanol (BDH Laboratory Supplies, Dubai, UAE) for 20 min at −20 °C, washed two times in 1× PBS and permeabilized in 0.3% (*v*/*v*) Triton X-100 (Fisher Scientific, Loughborough, UK) in PBS for 10 min. These were then incubated in 5% normal goat serum (NGS; Sigma-Aldrich)/PBS for 30 min. This was followed by an overnight incubation in a humid chamber with the primary antibody 1:100 anti-pSmad2/3 (sc-11769, Santa Cruz Biotechnology Inc., TX, USA) diluted in 0.5% NGS/PBS. Negative controls for the primary (0.5% NGS/PBS instead of the primary antibody) were used. Cells were then washed with PBS for 5 min followed by 1 h incubation in the presence of a rhodamine labelled secondary antibody (diluted 1:25; Jackson Immunoresearch Laboratories, Suffolk, UK), again in a humid chamber in the dark, washed again and then slides were mounted in vectashield anti-fading medium containing 4′, 6-diamidino-2-phenylindole (DAPI) (Vector Laboratories, Peterborough, UK) and visualized using an Axio Imager Zeiss (Jena, Germany) microscope. Nuclear to cytoplasmic expression of pSmad2/3 was determined by counting five randomly selected fields of stained cells imaged at 20× magnification. Data are representative of n = 3 experiments.

Cells were seeded in chamber slides and treated with CoCl_2_ or hypoxia for 48 h. Cells were then fixed in 4% paraformaldehyde (G1101, Servicebio, Wuhan, China) for 30 min at RT, washed two times in 1× PBS and permeabilized in 0.1% (*v*/*v*) Triton X-100 (Sigma-Aldrich) in PBS for 10 min. These were then blocked in 10% NGS/PBS for 1 h and incubated with the primary antibody against CXCR4 (1:100, AF6621, Beyotime Biotechnology, Nantong, China) diluted in 2% NGS/PBS overnight. Negative controls for the primary (2% NGS/PBS instead of the primary antibody) were used. Cells were then washed three times in 1× PBS and incubated with Alexa Flour 488-conjugated Goat anti-rabbit IgG secondary antibody or Alexa Flour 555-conjugated Goat anti-rabbit IgG secondary antibody (diluted 1:500; Thermo Fisher Scientific, Waltham, MA, USA) in the dark, washed again and were then counterstained with DAPI and visualized using a Leica SP8 confocal system.

### 2.9. Western Blot Analysis

Cells were harvested and treated with Western/IP lysis buffer (Beyotime Biotechnology, China) containing phenylmethyl sulonylfluoride (PMSF) and protease inhibitor cocktail set I (Millipore Corp, Burlington, MA, USA) at 4 °C for 20 min. Protein concentration was determined using the BCA protein assay kit (Beyotime Biotechnology, China). Proteins were separated using SDS-PAGE and subsequently transferred onto polyvinylidene difluoride (PVDF) membrane (Millipore Corp, USA). After blocking with 5% non-fat milk in TBST for 1 h, the membranes were incubated with primary antibodies against TGF-β1 (1:1000, 21898-1-AP, Proteintech, Wuhan, China), TGFBRII (1:1000, 66636-1-Ig, Proteintech, China), TGFBRI (1:1000, GB11271, Servicebio, China) and GAPDH (1:2000, ab181602, Abcam, Cambridge, UK) overnight at 4 °C. After washing with TBST buffer, the membranes were incubated with HRP-conjugated secondary antibody for 1 h at RT. The signal was detected with enhanced chemiluminescence (ECL) kit (Millipore Corp, USA).

### 2.10. Quantitative PCR (qPCR)

Total RNA was extracted using the RNeasy Micro kit (Qiagen, Hilden, Germany) as per manufacturer’s instructions. RNA aliquots were prepared by diluting the RNA samples to get a final concentration of 1000 ng/μL and reverse transcription was performed using the Bioline cDNA synthesis kit (Bioline, London, UK) for the preparation of cDNA as per manufacturer’s instructions. For qPCR analysis, 20 μL qPCR reactions were prepared in triplicates for each sample, containing 2 μL cDNA, 1 μL primer probes (TGFB-RI: Hs00610318_m1, TGFB-RII: Hs00559661_m1, TGF-β1: Hs00998129_m1, beta-actin: Hs01060665_g1, VEGF-A: Hs00900055_m1, CXCR4: Hs00607978_s1, (Life Technologies), 10 μL Brilliant II QPCR master mix with High ROX (Agilent Technologies LTD, Berkshire, UK) and DNase-free water (Promega, Wisconsin, USA) to make up a total volume of 20 μL. Non template controls (NTC) were used to ensure that all procedures were free of DNA contamination. All reactions were run in an Applied Biosystems Step One thermocycler. Relative expression levels for each gene of interest were calculated by normalizing the quantified gene of interest’s transcript level (threshold cycle; CT) to the CT value of the housekeeping gene b-actin using the 2^−ΔCT^ formula and multiplying by 100 to present the values as percentage of b-actin. Fold change was calculated using the 2^−ΔΔCt^ formula, where ΔΔCt = ΔCt of treated sample-ΔCt of untreated sample.

### 2.11. Determination for VEGF-A by ELISA Assay

Cell culture medium were centrifuged at 1500 rpm for 20 min to pellet potential contaminating cells and the clean supernatant was transferred into new E-tubes and stored at −80 °C. The Human VEGF-A ELISA Kit (BMS277-2, Thermo Fisher Scientific) was used to measure the expression of VEGF-A in the medium. The ELISA was determined according to the protocol provided by the supplier. The standard curve was determined by the recombinant VEGF-A provided in the kit. The absorbance of the samples was read at 450 nm.

### 2.12. Isolation of CoCl_2_ Treated and Control Cells for Single Cell Analysis

In order to determine the impact of CoCl_2_ at the transcriptional level on MCF7 SP cells we treated bulk cells with CoCl_2_ as described above, these were harvested at 48 h. Controls were cells from the same starting culture but cultured without CoCl_2._ We fluorescently barcoded one cell population (barcoding was reversed between CoCl_2_ treated and control cells over the several single cell experiments undertaken to determine that barcoding had no adverse effect on either cell population), for barcoding we used a CellTrace (TM) CFSE cell proliferation dye (Thermofisher, Waltham, MA, USA) as per manufacturer’s instructions. Then both cell preparations were stained using the SP assay described above and the two sorted SP populations were resuspended in PBS at a concentration of 250–300 cells/µL ready for single cell capture.

### 2.13. Single Cell Capture

Single SP cells were captured and pre-amplification performed using the C1 system (Fluidigm, San Francisco, CA, USA), as per manufacturer’s protocol. A 50:50 ratio of control to treated cell suspension was prepared at a concentration of 250–300 cells/µL PBS. Cells were separated using a C1 Single-Cell Preamp integrated fluidic circuit (size 10–17 μm) (Fluidigm). IFC priming, cell loading and lysis, reverse transcription and pre-amplification was then carried out using a range of kits including the C1 Single-Cell Auto Prep Reagent Kit (Fluidigm), Ambion Single Cell-to-CT qRT-PCR Kit (Thermo Fisher Scientific) and 20X TaqMan Gene Expression primers (Thermo Fisher Scientific). After loading, partitioned single cells were visualized under bright field and florescence using an Axiovert 200 M microscope (Zeiss). Following pre-amplification amplicons were transferred to a 96 well plate and stored at −20 °C.

### 2.14. Single Cell Gene Expression Analysis

This was carried out using a Biomark HD system (Data Collection software version 4.1.3) and IFC Controller HX (Fluidigm) following the manufacturer’s quick reference guide. Gene expression analysis was carried out using Dynamic Array Flex Six Gene Expression IFCs (Fluidigm). Assays were performed using the Flex Six Gene Expression Reagent Kit (Fluidigm) as per manufacturer’s instructions with 20× TaqMan Gene Expression primers (Thermo Fisher Scientific) and TaqMan Fast Advanced Master Mix (Thermo Fisher Scientific). Cells (both treated and untreated) where analysed from three separate cultures. After quality control 115 cells were included in the final analysis (52 treated and 64 control).

### 2.15. Statistics

Statistical analyses and graphical representation of results were performed using GraphPad Prism version 3 (GraphPad, San Diego, CA, USA) for analysis both, unpaired and paired two tailed student t-tests or column statistics were used. Data was considered statistically significant at *p* < 0.05.

## 3. Results

### 3.1. EMT Induction through TGF-β1, Hypoxia and CoCl_2_ Treatment

**Figure 1:**
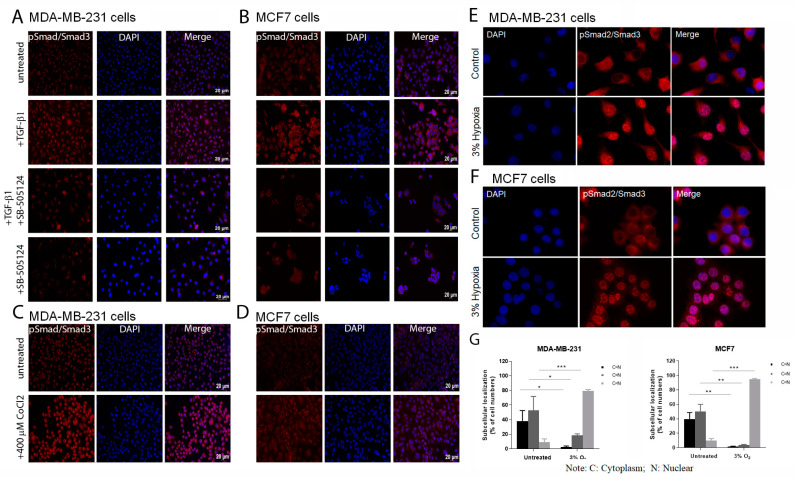
Immunocytochemical (ICC) analysis for pSmad2/3 expression following treatment with TGF-β1 or CoCl_2_ in both MDA-MB-231 and MCF7 cell lines. Increased expression of pSmad demonstrates activation of the TGF-β signalling pathway in both breast cancer cell lines upon treatment with 5 and 10 ng/mL TGF-β1, respectively, for 72 h compared to controls and untreated cells. Note the addition of 5 µM of SB-505124 in the presence or absence of TGF-β1 results in decreased pSmad expression in both cell lines (**A**) MDA-MB-231 (**B**) MCF7 cells. ICC images for pSmad2/3 staining showing activation of the TGF-β signalling pathway induced via exposure to 400 μΜ CoCl_2_ for 48 h in (**C**) MDA-MB-231 and (**D**) MCF7 cells or culture in 3% oxygen for 48 h in (**E**) MDA-MB-231 and (**F**) MCF7 cells. (**G**) Graphical representation of the proportion of cells showing either cytoplasmic (C) or nuclear (N) localization after hypoxia exposure; C < N, C = N, C > N. Note increased pSmad staining after treatment compared to that in negative control and untreated cells. Representative images from n = 3 experiments. Scale bar = 20 µm, pSmad expression = red, nuclei were stained with Dapi = blue. (**E**,**F**) Original magnification 40×. *p* values * *p* value < 0.05, ** *p* value < 0.005, *** *p* value ≤ 0.001.

MDA-MB-231 cells were treated with 5 ng/mL TGF-β1 for 3 days and then stained for pSmad expression (Figure 1A). Untreated cells showed low pSmad2/3 levels. Whereas exposure to TGF-β1 resulted in increased expression of pSmad2/3 which was reversed upon the addition of the TGFB-RI antagonist, SB-505124 with or without the presence of ΤGF-β1 (Figure 1A). In the MCF7 cell line, treatment with 5 ng/mL TGF-β1 was not sufficient to induce EMT while 10 ng/mL increased pSmad2/3. SB-505124 with and without TGF-β1 also reduced the levels of nuclear pSmad2/3 in the MCF7 cell line (Figure 1B).

To determine if CoCl_2_ exposure can also activate the TGF-β signalling pathway, the levels of pSmad2/3 protein levels in unfractionated MDA-MB-231 and MCF7 cells untreated and treated with 400 μΜ CoCl_2_ for 48 h were assessed. Treatment resulted in an increase in pSmad2/3 levels in both cell lines (Figure 1C,D).

Cells of both cell lines were also incubated in a hypoxic environment (3% oxygen for 24 h) and assessed for pSmad2/3 and again treatment resulted in an increase in pSmad2/3 levels (Figure 1E,F). In addition, after culture in 3% oxygen, both cell lines showed a greater proportion of cells with nuclear staining for pSmad2/3 (Figure 1G).

### 3.2. Assessment of TGF-β Receptor Expression and Endogenous TGF-β1 Production in MDA-MB-231 and MCF7 Cells

**Figure 2:**
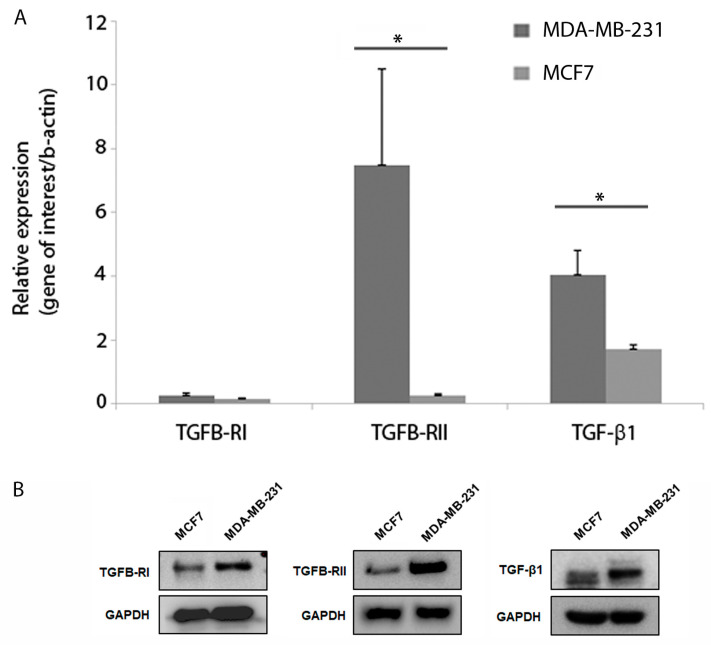
Assessment of TGF-β receptor expression and endogenous TGF-β1 production in MDA-MB-231 and MCF7 cells. (**A**) Graphical representation of qPCR data for TGFB-RI, TGFB-RII and TGF-β1 mRNA levels in MDA-MB-231 and MCF7 cells. TGFB-RI was not expressed at significantly different levels between the two cell lines n = 5, *p* = 0.33, however, TGFB-RII and TGF-β1 expression was significantly higher in the MDA-MB-231 than in the MCF7 n = 5, *p* = 0.04 and n = 3, 0.04, respectively. * *p* value < 0.05. (**B**) Expression of TGF-β1, TGFB-R1 and TGFB-RII protein was determined using Western blotting. Note expression is lower for all three proteins in the MCF7 cell line.

q-PCR analysis was used to measure mRNA expression of TGF-β1, TGFB-RI and TGFB-RII. There was no significant difference in expression of TGFB-R1 between the two cell lines (Figure 2A), while levels of TGF-β1 and TGFB-RII were significantly higher in the MDA-MB-231 than in the MCF7 cells (*p* = 0.04) (Figure 2A). In addition, endogenous expression of TGF-β1, TGFB-R1 and TGFB-RII protein was determined using Western blotting for both cell lines. Both cell lines expressed TGF-β1 and both receptors, with expression in all cases being lower in the MCF7 than the MDA-MB-231 (Figure 2B). Images of whole western blots are provided (Supplementary Figure S1).

### 3.3. Confirmation of the Induction of Hypoxia

**Figure 3:**
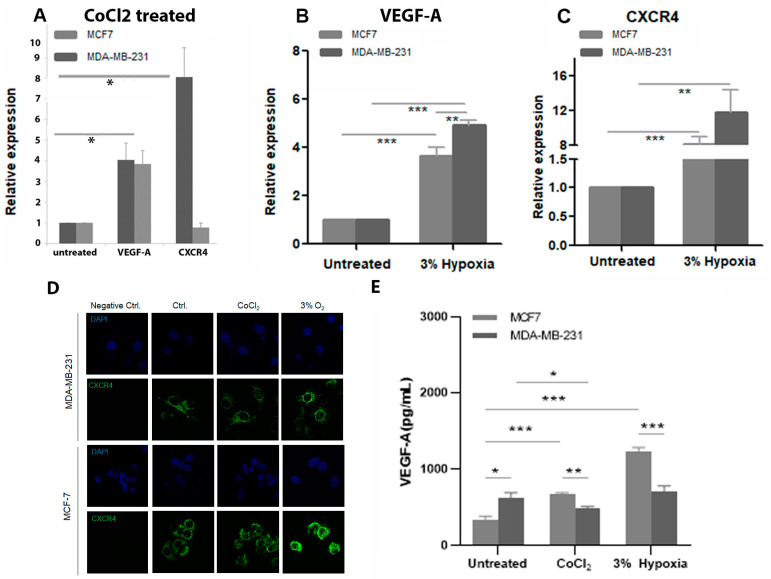
Gene and protein expression of VEGF-A and CXCR4 following treatment with CoCl_2_ or low oxygen culture. Graphical representation of qPCR data for mRNA expression levels of VEGF-A and CXCR4 in MDA-MB-231 and MCF7 cells treated with 400 μΜ CoCl_2_ for 24 h (**A**). Untreated cells are included as control. Bars represent an average of n = 3 individual experiments. Note the significant increase in VEGF-A expression (*p* = 0.02) and CXCR4 expression (*p* = 0.04) in the treated MDA-MB-231 versus the untreated. Following treatment by culture under 3% hypoxia note that there was a significant difference in expression of VEGF-A between both cell lines and their controls (n = 3, MDA-MB-231 *p* < 0.001, MCF7 *p* < 0.001) (**B**). This was also apparent between the hypoxia treated MCF7 and MDA-MB-231 with expression being significantly higher in the MDA-MB-231 (*p* = 0.007) (**B**). While for CXCR4 expression both cell lines showed significantly increased expression after hypoxia treatment compared to controls (n = 3, MDA-MB-231 *p* < 0.001, MCF7 *p* = 0.002) (**C**).To determine if either treatment led to modification of protein expression, cells of both cell lines were exposed to CoCl_2_ and hypoxia and stained for expression of CXCR4 (**D**). For both cell lines CXCR4 expression increased slightly under both conditions compared to controls. Representative images of n = 3, original magnification 40×. (**E**) VEGF-A protein expression following exposure to CoCl_2_ or low oxygen levels was determine by ELISA. In untreated cells, secreted levels of VEGF-A were higher in the untreated MDA-MB-231 compared to untreated MCF7 (*p* = 0.01). In the MCF7 cells both CoCl_2_ and hypoxia treatment increased secretion of VEGF-A (both *p* < 0.001). Whereas, in the MDA-MB-231 cells CoCl_2_ decreased secretion of VEGF-A (*p* = 0.04), and hypoxia had no effect. *p* values * *p* value < 0.05, ** *p* value < 0.005, *** *p* value ≤ 0.001.

To determine if CoCl_2_ induced hypoxic conditions in MDA-MB-231 and MCF7 cell lines, the levels of two hypoxia-responsive genes VEGF-A and CXCR4 before and after treatment with 400 μΜ CoCl_2_ for 24 h were measured (Figure 3A). Controls were untreated cells of both cell lines. VEGF-A and CXCR4 mRNA expression levels were increased significantly in the treated MDA-MB-231. The expression of VEGF also increased in the MCF7 treated cells compared to control, but this was not significant and CXCR4 expression was not increased. This was compared to expression of the above two hypoxia-responsive genes in both cell lines being cultured under hypoxic conditions (3% oxygen). This led to an increase in both VEGF-A and CXCR4 in both cell lines compared to controls (controls were cells incubated under normal TC oxygen levels for 24 h). The increase in VEGF-A levels was significantly different in both cell lines compared to controls (Figure 3B). While an increase in VEGF-A levels between hypoxia treated MDA-MB-231 and hypoxia treated MCF7 was observed with levels being higher in MDA-MB-231 (Figure 3B). CXCR4 expression was also increased significantly for both hypoxia-treated cell lines compared to controls (Figure 3C).

Next, we undertook ICC analysis of CXCR4 protein expression in both cell lines. Under both treatment conditions CXCR4 protein expression appeared to have increased slightly (Figure 3D). In addition, secretion of VEGF was assessed by ELISA, with levels increasing after both treatment with CoCl_2_ and culture at 3% oxygen in the MCF7 cell line compared to controls, but not in MDA-MB-231 (Figure 3E).

### 3.4. The Effect of TGF-β1 Treatment on SP Numbers

**Figure 4:**
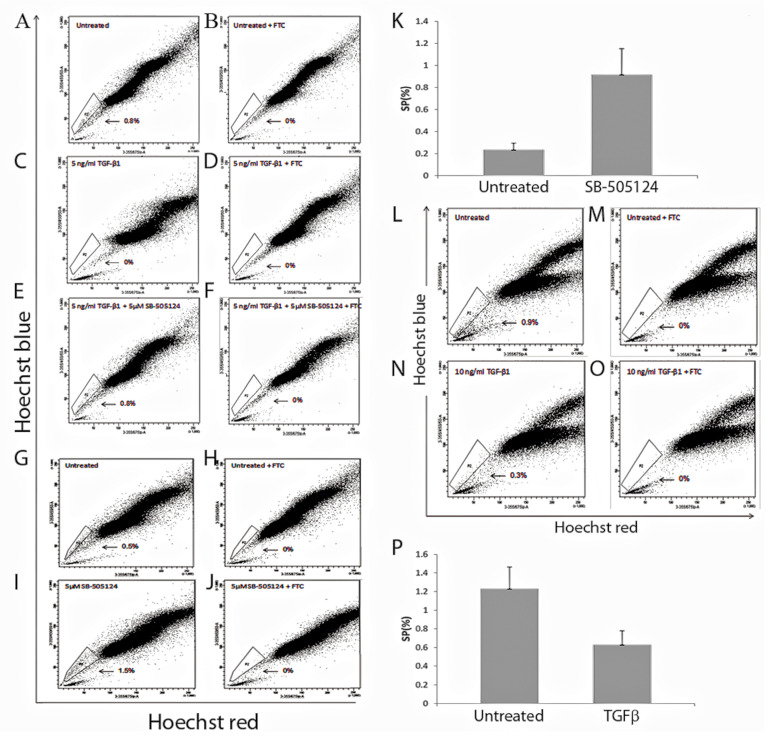
Impact of TGF-β1 treatment on SP cells of the MDA-MB-231 and MCF7 cell lines. Representative FACS images of SP analysis in MDA-MB-231 cells. (**A**) Untreated (**C**) Treated with 5 ng/mL TGF-β1 (**Ε**) Treated with 5 ng/mL TGF-β1 and 5 μΜ SB-505124. FTC, was used to confirm the SP phenotype in (**B**,**D**,**F**). Representative FACS images of SP cells from the same culture (**G**) 0.5% SP percentage in untreated cells, while in the presence of 5 μΜ SB-505124 only, this percentage increased to 1.5% (**I**). For both untreated and treated cells FTC was used as a control to confirm the SP phenotype (**H**,**J**). The SP percentage for both untreated and SB-505124 treated MDA-MB-231 are represented graphically in (**K**). Bars represent an average of n = 6 individual experiments, note the increase in SP percentage in the presence of SB-505124, *p* = 0.03. Representative FACS images of SP analysis in MCF7 cells (**L**) Untreated, (**N**) Treated with 10 ng/mL TGF-β1. FTC was used to confirm the SP phenotype in (**M**,**O**). The SP percentages for both untreated and TGFβ treated MCF7 are graphical represented (**P**). Bars represent n = 7 individual experiments, note the significant increase in MCF7 SP in the untreated group compared to the cells treated with TGF-β1, *p* = 0.03.

MDA-MB-231 cells were treated with 5 ng/mL TGF-β1 for 72 h and SP analysis was performed. Untreated cells contained an SP population (Figure 4A) that was completely depleted by TGF-β1 treatment (*p* = 0.04) (Figure 4C). The use of SB-505124 5 μΜ in the presence of TGF-β1 led to an increase in the SP percentage (0.8%) (Figure 4E), back to the level seen in the untreated cells (0.8%) (Figure 4A). Addition of SB-505124 alone without TGF-β1 treatment further increased the SP percentage (*p* = 0.03) (Figure 4I) from that of control (Figure 4G). This is represented graphically (Figure 4K). For both untreated and treated cells, the ABCG2 specific inhibitor fumitremorgin C (FTC) was used as a control to confirm the SP phenotype (Figure 4B,D,F,H,J).

Bulk MCF7 cells were treated with 10 ng/mL TGF-β1, (use of 5 ng/mL did not effectively eliminate the SP population) for 3 days and SP analysis was performed. A significant reduction was observed between the untreated SP (Figure 4L) and the treated SP (Figure 4N) populations. Statistical analysis (based on the result of n = 7 biological replicates) revealed a significant reduction (*p* = 0.03), of the SP numbers when compared with untreated SP (Figure 4P). The SP phenotype was confirmed for all groups by the addition of FTC (Figure 4M,O). Treatment with SB-505124 was not possible in the MCF7 cells, as it caused cell death.

### 3.5. The Effect of CoCl_2_ Treatment on SP Numbers

**Figure 5:**
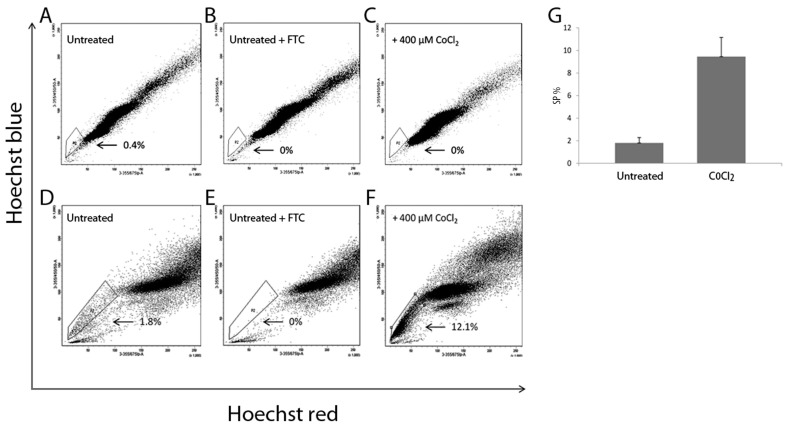
Assessment of the impact of CoCl_2_ treatment on SP cells of the MDA-MB-231 and MCF7 cell lines. Representative FACS images of SP analysis in MDA-MB-231 (**A**) Untreated (**C**) Treated with 400 μM CoCl_2_ for 48 h alone. Note the SP population was eliminated, this represents n = 4, *p* = 0.03. FTC was used to confirm the SP phenotype in (**B**). Representative FACS images of SP analysis in MCF7 cells (**D**) Untreated (**F**) Treated with 400 μM CoCl_2_ for 48 h alone. FTC was used to confirm the SP phenotype (**E**). (**G**) Graphical representation of MCF7 flow cytometry data, percentages of SP cells in untreated and treated with 400 μM CoCl_2_. Bars represent an average of n = 4 individual experiments, *p* = 0.03.

Both cell lines were treated with 400 μM CoCl_2_ for 48 h followed by SP analysis. Representative images show the presence of SP cells in the untreated MDA-MB-231 and MCF7 cell lines (Figure 5A,D). After treatment of MDA-MB-231 the SP phenotype was lost (n = 4, *p* = 0.03) (Figure 5C), while MCF7 treatment resulted in a significant increase in the SP percentage (n = 4, *p* = 0.03) (Figure 5F). These data are represented graphically in Figure 5G. The SP phenotype was confirmed by the addition of FTC to untreated MDA-MB-231 (Figure 5B) and MCF7 cell lines (Figure 5E).

### 3.6. The Effect of CoCl_2_ Treatment on Mammosphere Formation

**Figure 6:**
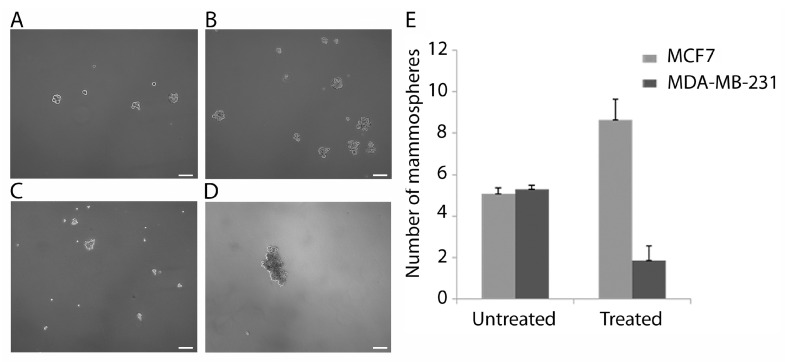
Analysis of impact of CoCl_2_ treatment of the mammosphere forming potential of MCF7 and MDA-MB-231 cells. Representative brightfield images of mammospheres from untreated MCF7 (**A**) and MDA-MB-231 (**C**) at day 9, compared with CoCl_2_ treated cells of MCF7 (**B**) and MDA-MB-231 (**D**). Images were taken at 5× magnification and a 100 µm scale bar is shown. Graphical representation of mammosphere forming assay data, number of mammospheres were counted using brightfield microscopy on day 9 of culture for treated and untreated cells (**E**). Bars represent an average of n = 3 individual experiments for both cell lines. Note the increase in mammosphere forming potential in the treated MCF7 compared to untreated *p* = 0.02. Note the reduced mammosphere forming potential of the treated MDA-MB-231 compared to untreated control *p* = 0.008.

Following the different response of SP cells in the MCF7 and MDA-MB-231 cell lines to CoCl_2_ treatment, the mammosphere forming potential of both cell lines treated with CoCl_2_ was also determined. Both cell lines were capable of mammosphere formation. MCF7 (Figure 6A) and MDA-MB-232 (Figure 6C). However, following CoCl_2_ treatment the number of mammospheres was significantly increased in MCF7 (*p* = 0.002) (Figure 6B,E) but significantly decreased in the MDA-MB-231 cells (*p* = 0.008) (Figure 6D,E).

### 3.7. The Effect of Tamoxifen and CoCl_2_ Treatment on the MCF7 SP Cells

**Figure 7:**
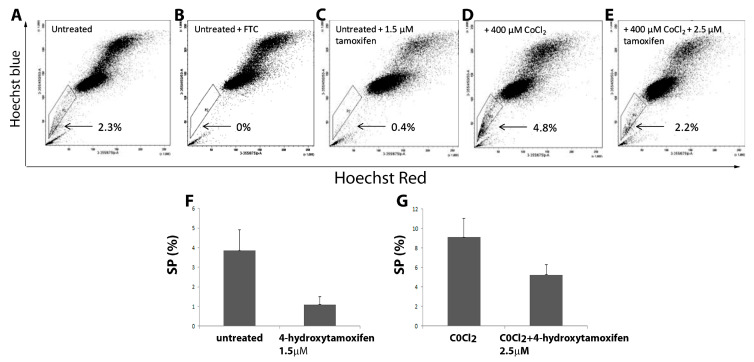
Analysis of the impact of CoCl_2_ and drug treatment on MCF7 SP cells. Representative FACS images of SP analysis of MCF7 cells treated with Tamoxifen (Tam) alone or in combination with CoCl_2_. (**A**) Untreated (**B**) Untreated with the addition of FTC to confirm the SP phenotype. (**C**) Treated with 1.5 μM Tam for 72 h alone. (**D**) Treated with 400 μM CoCl_2_ for 48 h alone. (**E**) Treated with 2.5 μM Tam for 72 h and 400 μM CoCl_2_ for 48 h. (**F**) Graphical representation of flow cytometry data showing percentages of MCF7 SP cells both untreated and treated with 1.5 μM Tam. Bars represent an average of n = 4 individual experiments. Note the significant decrease in SP when comparing untreated to Tam treated cells, *p* = 0.04. (**G**) Graphical representation of percentages of MCF7 SP cells treated with 400 μM CoCl_2_ alone and treated with 400 μM CoCl_2_ and 2.5 μM Tam. Bars represent an average of n = 5 individual experiments. Note the significant decrease when CoCl_2_ treated cells are treated with 2.5 μM Tam *p* = 0.03.

To determine the impact of Tamoxifen (Tam) on MCF7 SP we compared untreated MCF7 cells (Figure 7A) with cells treated with 1.5 μΜ Tam alone (Figure 7C) and with CoCl_2_ treatment alone (Figure 7D). Representative FACS images show untreated cells had a SP of 2.3% that was significantly decreased in cells treated with 1.5 μΜ Tam alone (*p* = 0.04) (Figure 7C,F) compared to control (Figure 7A). When treated only with 400 μM CoCl_2_ the percentage of SP cells increased above that of control (Figure 7D). Addition of 1.5 μΜ Tam to the CoCl_2_ treated cells did not successfully deplete the MCF7 SP. However, a higher concentration of Tam (2.5 μΜ) was capable of significantly reducing the SP numbers in cells exposed to CoCl_2_ (*p* = 0.03) (Figure 7E,G). The SP phenotype was again confirmed using the addition of FTC to untreated MCF7 (Figure 7B).

### 3.8. The Impact of CoCl_2_ Exposure on the Transcriptome of MCF7 SP Cells

**Figure 8:**
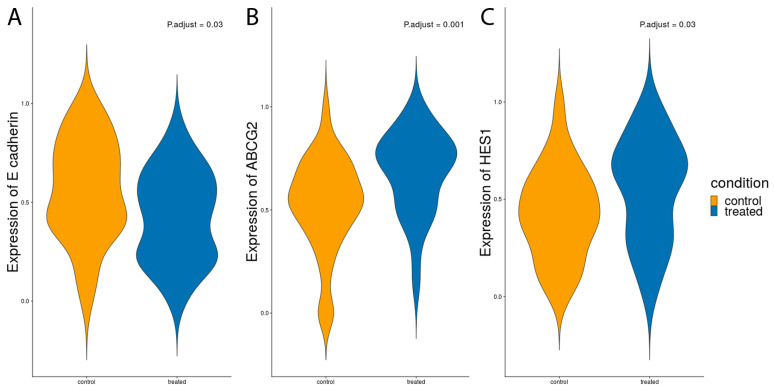
Transcriptional analysis of MCF7 CoCl_2_ treated and untreated SP cells. Violin Plots were used to represent expression levels of mRNA for E-cadherin (**A**) ABCG2 (**B**) and HES1 (**C**). Note decreased expression of E-cadherin in the treated cells but an upregulation of ABCG2 and HES1 following treatment with CoCl_2_.

Using a single cell approach, we determined if exposure to CoCl_2_ influenced the expression of genes known to be stem cells markers, markers for proliferation, migration, invasion, drug resistance, EMT and MET in the MCF7 SP cells. Only three of these genes were significantly different in terms of expression. Violin plots show the distribution of transcripts for e-cadherin, ABCG2 and HES1 in CoCl_2_ treated and untreated MCF7 SP cells (Figure 8). Note that e-cadherin was significantly reduced following exposure to CoCl_2_ compared to untreated MCF7 SP cells (Figure 8A), whereas following CoCl_2_ treatment MCF7 SP showed an increase in ABCG2 expression (Figure 8B) and an increase in HES1 (Figure 8C) expression compared to untreated MCF7 SP, both of which were significant. None of the other genes examined showed significant changes between the treated and untreated cells.

## 4. Discussion

The role of TGF-β and hypoxia in EMT and CSC regulation in breast cancer has become of major interest. This pathway in breast cancer has a dual role, in early stages of the disease it has a tumour suppressive activity, while at later stages it promotes tumour progression. The impact of TGF-β driven EMT on several BCSCs populations has been studied previously, and leads to the enhancement in some, [6] and to the reduction in others [11].

In this study, we used the MDA-MB-231 and MCF7 breast cancer cell lines in order to study the role of EMT induced by TGF-β and hypoxia on the regulation of SP cells.

The use of the hypoxia mimetic CoCl_2_ was validated against low oxygen culture conditions by assessment of expression levels of two hypoxia responsive genes. There are advantages and limitations to both methods. Maintaining cells in low oxygen conditions can be difficult due to the need to remove cells for maintenance, and in the case of SP cells the need to be sorted for further analysis could lead to fluctuations in hypoxia levels, which could impact on experimental outcomes. In addition, neither cell line tolerated oxygen concentrations lower than 3% and CoCl_2_ treatment can be maintained in culture media for longer periods of time.

The activation of the TGF-β signalling pathway was confirmed by exposure of both cell lines to TGF-β, CoCl_2_ and low oxygen in both cell lines as shown by the significant increase in pSmad2/3 levels in the treated samples.

We then investigated the effect of TGF-β treatment on the SP population. This showed complete loss of the MDA-MB-231 SP phenotype [24] and a significant reduction in the MCF7 SP population. Our MCF7 SP data is consistent with another study that demonstrated a significant decrease in the MCF7 SP which was reversed when TGF-β treatment was withdrawn [11]. Our analysis of the responsiveness of the MCF7 and MDA-MB-231 cells lines to TGF-β1 treatment revealed differences between them. For the MDA-MB-231 the loss of SP following treatment could be reversed by the use of SB-505124, which resulted in the re-appearance of the SP population [24]. Moreover, treatment of MDA-MB-231 cells with SB-505124 in the absence of exogenous TGF-β1 further enhanced the SP phenotype [24], suggesting autocrine TGF-β1 production is responsible for reducing the SP numbers in this case. MCF7 cells did not survive the use of the SB-505124 inhibitor which we suggest was due to internalization of TGF-β1 and weak TGF-β signalling.

The negative regulatory role of TGF-β-driven EMT on SP cells has been shown in several other cell types. SP cells exhibited a higher tumorigenicity in vivo compared to the NSP cells from diffuse-type gastric carcinoma cell lines. This was however, reduced when cells were pre-treated with exogenous TGF-β before being injected into nude mice. Further, knock-down of Smad4 did not result in repression of ABCG2 expression in response to TGF-β, indicating that these effects were due to the TGF-β/Smad dependent pathway [10]. SP cells from some pancreatic cell lines have also been reported to be more responsive to EMT changes induced by TGF-β in comparison to NSP cells [25]. TGF-β treatment also caused a reduction in SP in hepatic stellate LX2 cells [26]. In contrast, in the human gallbladder cancer cell line GBC-SD, following treatment the SP cells showed upregulated ABCG2 expression and increased drug resistance [27].

We also examined the mRNA expression of TGFB-RI and II and TGF-β1 in both cell lines and found that RII and TGF-β1 levels were significantly higher in the MDA-MB-231 cells, while RII expression was low in MCF7 cells. These results are consistent with published findings that MCF7 cells lack expression of RII, restoration of which led to the cells becoming less tumorigenic [28]. It has been proposed that MCF7 cells are resistant to the inhibitory effects of TGF-β [29] due to the fact that RII expression (low in MCF7) can be saturated by TGF-β1 and therefore it reaches the cells’ maximal autocrine TGF-β activity more easily [30].

Using single cell analysis of MCF7 SP cells from the same cultures treated with and without CoCl_2_, we also observed a significant increase in ABCG2 and HES1 expression and a decrease in e-cadherin in CoCl_2_ treated MCF7 SP cells compared to control, suggesting activation of the EMT pathway and increased drug resistance in the treated MCF7 SP. HES1 has also been reported to promote EMT, contribute to metastasis and contribute to increased multidrug resistance of cancer cells [31].

The impact of CoCl_2_ on mammosphere formation in both cell lines followed a similar pattern to that of the treated SP cells, with the number of mammospheres being significantly reduced in MDA-MB-231 and significantly increased in MCF7 cells. These findings are consistent with another study that reported that estrogen receptor α (ERα) status affects the responsiveness of CSCs to hypoxia both in vitro and in vivo [32]. In addition, they reported that ERα was essential for the induction of hypoxia-related changes, with mammosphere forming efficiency being increased in ERα-positive primary samples and cell lines and decreased in ERα-negative primary samples and cell lines in response to hypoxia. These effects were reversed on addition of 4-hydroxytamoxifen, confirming that they were due to the activation of the ERα pathway [32].

There is accumulating evidence to support the crosstalk between the TGF-β and estrogen-signalling pathways. ER- breast cancer cell lines have been found to express receptors for TGF-β, while ER+ cell lines are characterized by low/no levels of these [8]. Furthermore, two distinctive stem cell populations were identified in primary human breast cancer cells: CD44-, CD24+, ER+, TGFB-RII- or CD44+, CD24-, ER-, TGFB-RII+ with the latter only being able to undergo EMT due to TGF-β treatment [9]. Therefore, ERα expression seems to play a pivotal role in the EMT response of breast CSCs. TGFB-RII receptor expression is inversely correlated with ERα expression in ERα positive breast cancer patients leading to weak TGF-β signalling. This could explain the observation that SP cells from the MCF7 cell line in our study were less responsive to the induction of EMT upon TGF-β exposure and were more prone to increase in CoCl_2_ culture conditions.

HIF-1α seems to positively regulate the expression of ABCG2. However, ERα also promotes the activation of the Notch signalling pathway by activating Notch1 and Jagged1, resulting in the expression of stem cells markers and HIF-1α [33], which can further increase ABCG2 expression. However, in ERα negative patients the TGF-β pathway is intact and thus ABCG2 expression is repressed. Further links between Erα, ABCG2 and cancer stem cells still need to be determined.

It has been reported that in MCF7 cells ERα induces pSmad2/3 degradation by forming a complex with it and ubiquitin ligase Smurf [34]. Similarly, when the ERα gene was introduced into MDA-MB-231 cells it resulted in decreased tumour formation both in vitro and in vivo. These effects were reversed in cells overexpressing ERα and a constitutively active form of Smad2. Consequently, ERα was proposed as a negative regulator of the TGF-β/Smad dependent signalling pathway in later stages of breast cancer, although estrogen exposure drives tumour progression in early stages [35].

To determine if CoCl_2_ had an impact on drug resistance in MCF7 SP we looked at the impact of tamoxifen treatment in combination with CoCl_2._ We observed that with combined treatment using a tamoxifen dose of 2.5 µM, the MCF7 SP cell numbers were reduced compared with use of CoCl_2_ alone. We opted to use tamoxifen as it is known to target ER+ breast cancer cells and MCF7 cells are predominantly ER+. As the combined treatment did not deplete the MCF7 SP it could be that hypoxia has a protective effect on the SP cells [20,21] or be due to the presence of a tamoxifen resistant subpopulation of MCF7 cells that have been reported to have cytotoxic resistance to tamoxifen [36].

## 5. Conclusions

We have demonstrated that EMT impacts on SP cells in the MDA-MB-231 and MCF7 breast cancer cell lines. We have shown that the TGF-β driven EMT effect is similar in both cell lines causing a loss of the SP cell phenotype in the MDA-MB-231 [24] and a reduction in MCF7 SP cells. However, the two cell lines showed diverse effects in response to hypoxia, with MDA-MB-231 SP being reduced and MCF7 SP increased. The diversity of the response of the SP in these two cell lines is linked to the levels of TGFβ receptor expression and ER status. MCF7 cell are ER+ and express low levels of TGFB-RII whereas MDA-MB-231 cells are ER- and express higher levels of TGFB-RII. The diversity of these effects may also reflect the differences in the responsiveness of the SP populations contained in patients with different breast cancer subtypes. We believe that this observation is of great clinical significance since breast cancer patients belonging to the triple negative or hormonal non-responsive subgroup have limited therapeutic options and anti-hormonal treatment cannot be used in these cases. Most importantly, these patients’ clinical condition and worse prognosis may mainly be due to the higher prevalence of the SP phenotype, which has been significantly associated with this particular breast cancer subtype [5]. Therefore, we expect that these patients may benefit from the TGF-β tumour suppressive actions and the inhibitory role of this pathway on the SP population, as opposed to patients with low or absent TGF-BRII expression.

## Data Availability

Data is contained within the article.

## Supplementary Materials

The following supporting information can be downloaded at: https://www.mdpi.com/article/10.3390/cancers15041108/s1, Figure S1: The original western bolt images of Figure 2B.
