# Supplementary material for: Hypoxia-Driven TGFβ Modulation of Side Population Cells in Breast Cancer: The Potential Role of ERα
Source: Cancers (Basel). 2023 Feb 9;15(4):1108. doi: 10.3390/cancers15041108 (PMC9954173; doi:10.3390/cancers15041108)
Supplement: Supplementary file 1 [file cancers-15-01108-s001.zip › cancers-2120226-supplementary.pdf]

Original data for WB

For TGF-β1

GAPDH

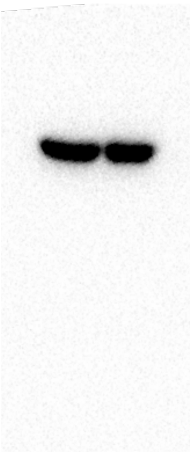

TGF-β1

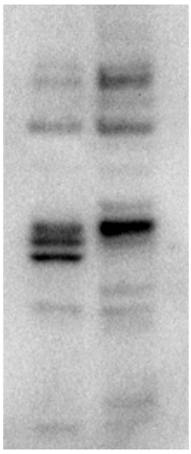

For TGFB-RI

GAPDH

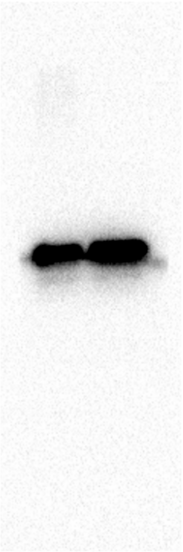

TGFB-RI

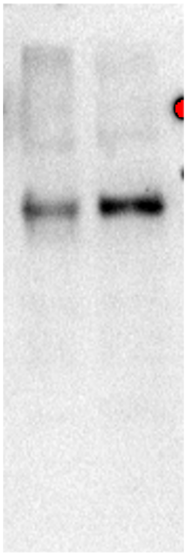

For TGFB-RII

GAPDH

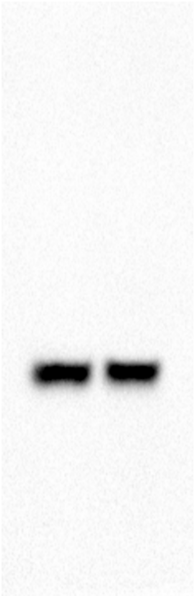

TGFB-RII

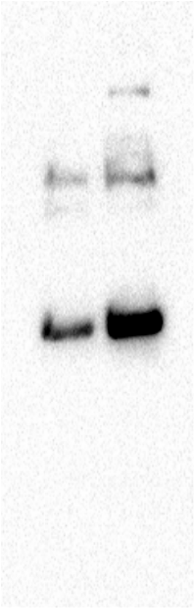

Figure S1. The original western bolt images of Figure 2B.
